# Acoustofluidic scanning fluorescence nanoscopy with large field of view

**DOI:** 10.21203/rs.3.rs-3069123/v1

**Published:** 2023-06-26

**Authors:** Geonsoo Jin, Joseph Rich, Jianping Xia, Neil Upreti, Chenglong Zhao, Tony Jun Huang

**Affiliations:** 1 Thomas Lord Department of Mechanical Engineering and Materials Science, Duke University, Durham, NC 27708, USA; 2 Department of Biomedical Engineering, Duke University, Durham, NC 27708, USA; 3 Department of Physics, University of Dayton, 300 College Park, Dayton, OH 45469, USA; 4 Department of Electro-Optics and Photonics, University of Dayton, 300 College Park, Dayton, OH 45469, USA

**Keywords:** Scanning nanoscope, Acoustics, Acoustofluidics, Super-resolution imaging, Fluorescence amplification

## Abstract

Nanoscale fluorescence imaging with a large-field view is invaluable for many applications such as imaging of subcellular structures, visualizing protein interaction, and high-resolution tissue imaging. Unfortunately, conventional fluorescence microscopy has to make a trade-off between resolution and field of view due to the nature of the optics used to form an image. To overcome this barrier, we have developed an acoustofluidic scanning fluorescence nanoscope that can simultaneously achieve superior resolution, a large field of view, and enhanced fluorescent signal. The acoustofluidic scanning fluorescence nanoscope utilizes the super-resolution capability of microspheres that are controlled by a programable acoustofluidic device for rapid fluorescent enhancement and imaging. The acoustofluidic scanning fluorescence nanoscope can resolve structures that cannot be achieved with a conventional fluorescent microscope with the same objective lens and enhances the fluorescent signal by a factor of ~5 without altering the field of view of the image. The improved resolution with enhanced fluorescent signal and large field of view *via* the acoustofluidic scanning fluorescence nanoscope provides a powerful tool for versatile nanoscale fluorescence imaging for researchers in the fields of medicine, biology, biophysics, and biomedical engineering.

## INTRODUCTION

Fluorescence microscopy has become an indispensable technique in the fields of biology and medicine^1^ with applications ranging from microscale imaging of live cells to nanoscale imaging of DNA sequencing protocols.^2,3^ However, due to the structure of the objective lens in conventional fluorescence microscopy, a trade-off is made in terms of the resolution and field of view. A higher-resolution image from a conventional fluorescence microscope can be achieved by using a higher magnification (typically also with a higher numerical aperture) objective lens, but it is typically at the cost of a reduced field of view. One effective approach to solve the issue of increasing resolution while maintaining a large field of view is through utilizing scanning dielectric microspheres.^4–13^ When the dielectric microsphere has a higher refractive index than its outer medium, the propagated light is focused from the inside of the microsphere, and a highly localized electromagnetic beam is generated near its surface, a phenomenon known as the photonic nanojet, that allows for super-resolution imaging below the diffraction limit.

The photonic nanojet has been utilized to enhance the resolution of both white-light^14–25^ and fluorescent microscopic imaging.^26–28^ For example, an optical fiber probe was combined with a microsphere for the manipulation and detection of individual sub-100 nm fluorescence nanoparticles.^26^ Moreover, a 20 nm fluorescence nanoparticle was also detected by a microsphere array in a microfluidic manner.^27^ A semi-opened microwell on a microsphere also captured target samples and amplified the fluorescence signal by the photonic nanojet effect.^28^ However, these studies have a limited detection area due to the static microsphere imaging or fixed microsphere condition. By incorporating a dynamic scanning element, such as an AFM cantilever,^29,30^ mechanical stage movements,^31,32^ optical tweezer methods,^33^ or acoustofluidics,^34,35^ both high resolution and a large field of view can be achieved. Of these aforementioned methods, acoustofluidic manipulation is advantageous due to its programable process, vast particle size manipulation range, and contactless manipulation nature.^36–53^ Recently, we demonstrated that acoustically driven microspheres could act as scanning super-lenses to rapidly and simultaneously achieve a large field of view and a high resolution in a white-light microscope.^34,35^ However, its application to fluorescence microscopy is yet to be explored.

In this article, we introduce an enhanced acoustofluidic scanning nanoscope for fluorescence imaging and amplification. Under the same imaging conditions, the acoustofluidic fluorescent scanning nanoscope can distinguish structures that cannot be resolved from a conventional fluorescent microscope under the same objective lens, and enhance the fluorescent signal by a factor of ~5 without altering the field of view of the image. With these features, the acoustofluidic scanning fluorescence nanoscope achieves rapid and superior fluorescence imaging with a large field of view that could be utilized in the fields of biology, chemistry, materials science, engineering, and medicine.

## RESULTS AND DISCUSSION

### Configuration of the acoustofluidic scanning fluorescence nanoscope

**Fig. 1.**
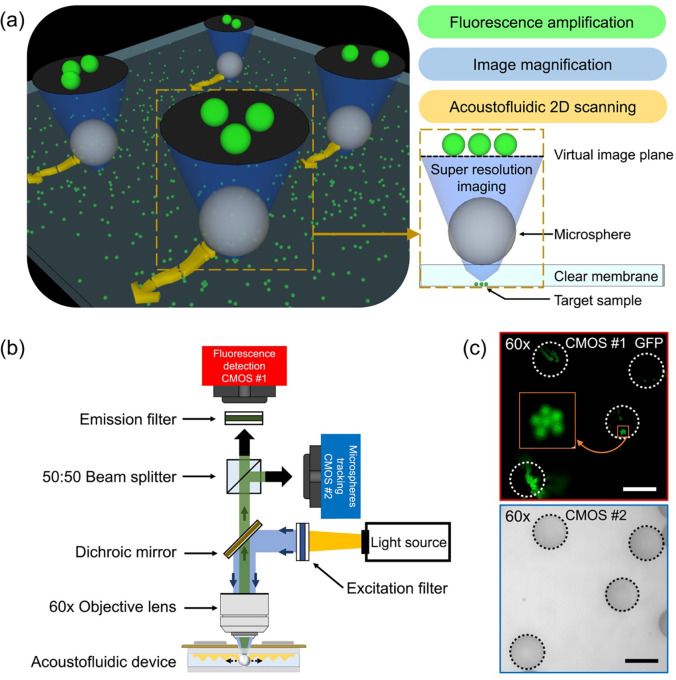
Mechanism of the acoustofluidic scanning fluorescence nanoscope. (a) 3D schematic of the system. A hard PDMS membrane on the target sample helps achieve the desired focal distance and demonstrate high-resolution images, as shown by the yellow box in the 2D schematic on the right. (b) Schematic of the optical setup. A 50:50 beam splitter delivered images into two different cameras, for both fluorescent detection (Camera #1, red box) and microsphere tracking (Camera #2, blue box). (c) Experimental results of enhanced fluorescent amplification of 500 nm fluorescent nanoparticle images (Camera #1, red box) through microspheres and microsphere particle tracking (Camera #2, blue box). Camera #2 focused on the center of the microspheres, as shown in the blue box. Only camera #1 was connected to an emission filter. Scale bars are 20 μm.

Figure 1(a) shows the 3D schematic figure of the acoustofluidic scanning fluorescence nanoscope. Super-resolution imaging is achieved when a microsphere was placed on the target sample as shown in the yellow dotted box in Figure 1(a). The sample consists of fluorescent nanoparticles that are drop-cast on a cover glass. The fluorescent particles are then covered by a thin layer of PDMS film to lock their positions on the cover glass and avoid drifting in the imaging process. A large field-of-view image is achieved by stitching the super-resolution image from each scanning microsphere. The scanning of microspheres is achieved by activating a propagating acoustic wave following the same method as we used before^34,35^ or counter-propagating acoustic waves that is demonstrated in this work. The advantage of using counter-propagating acoustic waves is that we can easily control the direction of the scan, which could not be achieved with a propagating acoustic wave.

Figure 1(b) shows the optical configuration of the system. Detailed specifications of the required components can be found in the experimental section. A white light source combined with a blue bandpass filter was used to illuminate the sample, which was passed through a dichroic mirror and focused through a 60x objective lens. The green fluorescent light from the green fluorescent nanoparticle was collected on a CMOS camera (shown as a red camera #1 in Figure 1(b)) through the same objective lens combined with a green bandpass filter (denoted as emission filter in Figure 1(b)). A 50:50 beam splitter was placed in the light pathway to add a second camera (shown as a blue camera #2 in Figure 1(b)) in the system without the emission filter in order to track the position of each microsphere. The position of the two cameras (#1 and #2) are adjusted in a way so that both the sample and the microsphere can be imaged simultaneously on camera #1 and camera #2, respectively. The red box in Figure 1(c) shows the fluorescence image of 500 nm fluorescence nanoparticles on camera #1 that are imaged through four microspheres. The four white dashed circles indicate the boundary of the four microspheres. Note that the four microspheres are invisible in the fluorescence image on camera #1. In contrast, they can be clearly imaged on camera #2 as shown in the blue box in Figure 1(c). The images of the microspheres on camera #2 are critically important for the determination of the exact position of each fluorescence image from each microsphere so that they can be stitched correctly to form the final large field-of-view image. The position of each microsphere can be obtained from the image on camera #2 by using a circle-finding algorithm in the image process. This position information from camera #2 can be assigned to the fluorescence image on camera #1. As a result, this dual-camera configuration allows us to form a high-resolution fluorescence image with a large field-of-view by precisely stitching the fluorescence images from each microsphere.

### Improve resolution and enhance the fluorescent signal with a microsphere

**Fig. 2.**
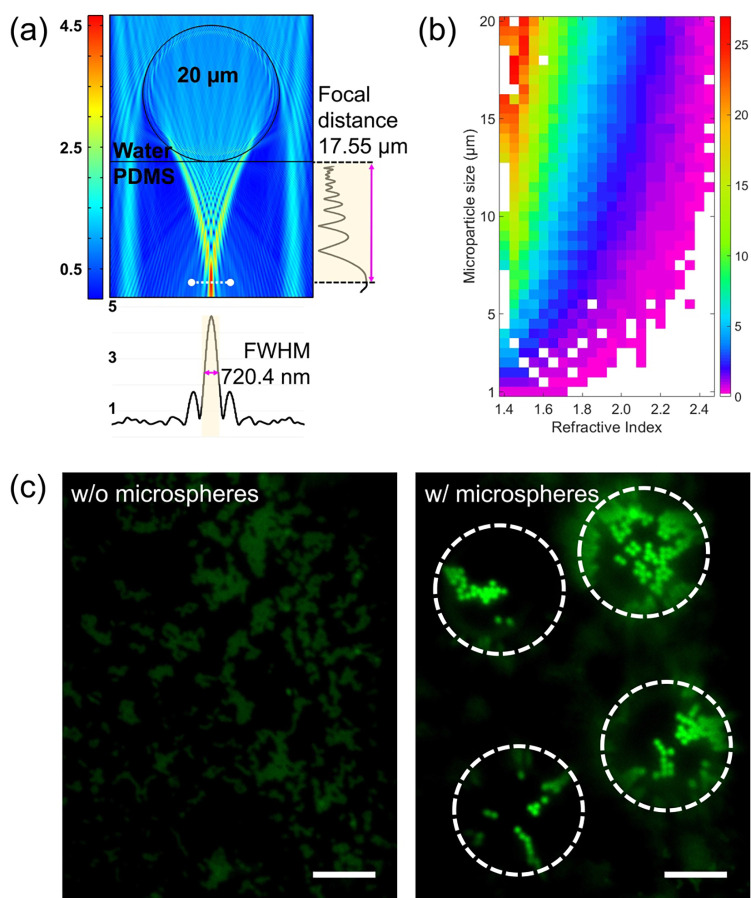
Simulation and experimental result of the photonic nanojets. (a) Finite element method (FEM) simulation result of a 20 μm polystyrene microsphere (n=1.58) in water (n=1.33) with a hard PDMS (n=1.41) film on the bottom as outer medium conditions. The vertical graph shows the focal distance from the bottom of the microsphere to the PDMS membrane. The horizontal graph represents the focused photonic nanojets and its full-width half-maximum. (b) FEM simulation result of the focal distance map as a function of the microsphere diameter and refractive index. The red color (25 μm focal distance) to purple color (3 μm focal distance) showed each focal distance depending on the size of the microsphere and refractive index. (c) Microscopic images of the microsphere magnification capability. The left panel is shown without microsphere condition. The right panel shows microsphere magnification at the same regions of interest as seen in the left panel. The target sample was 500 nm green fluorescence nanoparticles and thickness of the hard PDMS membrane was 7 μm. The scale bar is 10 μm.

Figure 2(a) shows the simulated electric field distribution of a 20 μm polystyrene microsphere (refractive index n=1.58) *via* finite element methods. The microsphere sits on a hard PDMS (n=1.41) with a surrounding medium of water (n=1.33) as that in the experiment. Light with a wavelength of 488 nm is used to excite the green fluorescence. The simulation confirmed that the light is well focused by the microsphere to a spot with a full width at half maximum (FWHM) of 720 nm and a distance of 17 μm away from its surface (defined as its focal length) as shown on the vertical graph in Figure 2(a). The focal length of a microsphere can be changed by choosing microspheres with a different size or refractive index. Figure 2(b) shows the color map of focal length as a function of the microsphere’s diameter and refractive index, which can be used as a guideline to select the right microsphere for the desired focal length. Changing the focal length of a microsphere changes the position of its virtual image from the microsphere, which needs to be compensated by adjusting the position of the objective lens for clear imaging.

Figure 2(c) shows the experimental fluorescent images of the same sample without and with a microsphere, respectively. The sample consists of aggregated fluorescence nanoparticles (500 nm in diameter) that are sandwiched between a 7 μm-thick PDMS film and a glass substrate (see experimental section). The two images shown in Figure 2(c) are taken in the same region of interest and settings such as light intensity and camera exposure time. The presence of the microsphere leads to both increased resolution of the system and enhanced fluorescent signals.

**Fig. 3.**
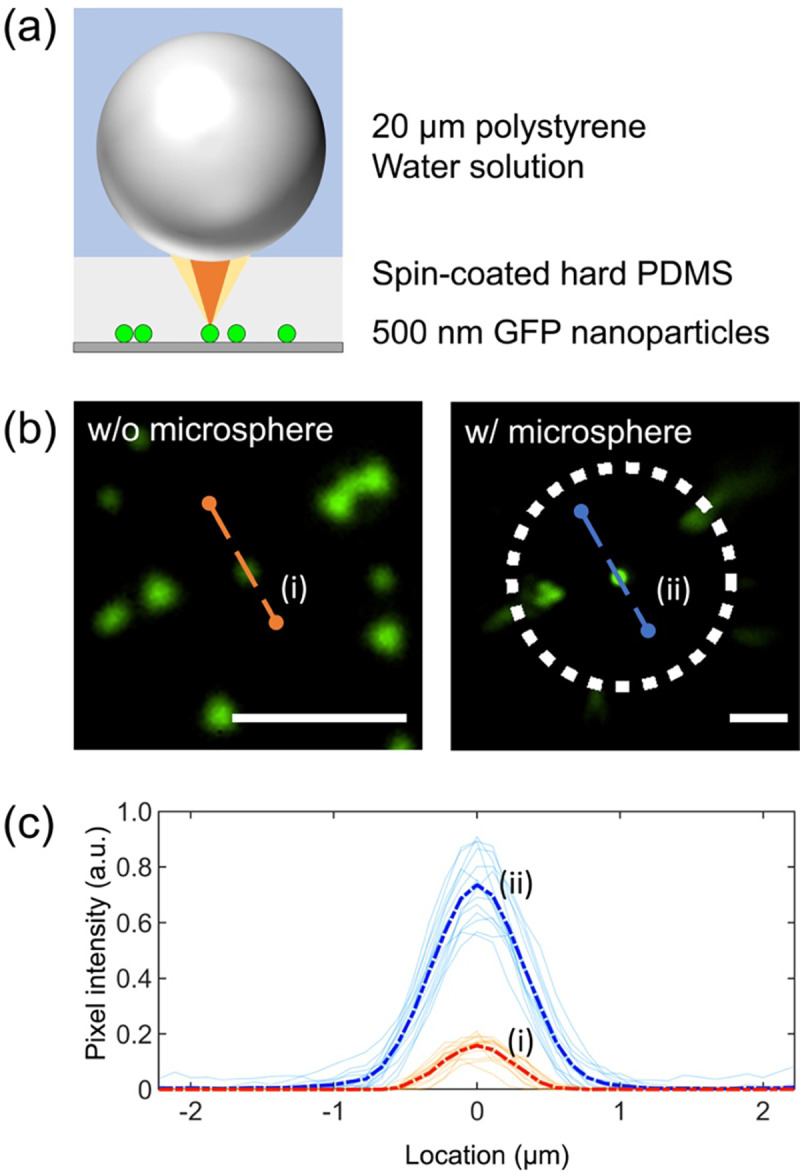
Fluorescence signal amplification with microspheres. (a) Schematic of fluorescence amplification with microsphere imaging. 500 nm green fluorescence nanoparticles were spread on the glass substrate. Then, a hard PDMS membrane was applied by a spin-coating process. 20 μm polystyrene microspheres and a deionized water solution were applied on the target sample. (b) Microscopic images of microsphere magnification and fluorescence amplification in a 7 μm thickness of hard PDMS. Since the microsphere magnified image size is bigger than that without microspheres, we represented the different magnification of each row of images with the different length of the scale bars. Scale bar is 5 μm. (c) Fluorescence amplification profile comparison under conditions without and with microspheres. The blue and red profile showed an average pixel profile intensity of 15 samples, as shown by without microsphere (i) and with microsphere (ii) conditions in Figure 3(b).

The fluorescence amplification results are illustrated in Figure 3. Figure 3(b) shows fluorescent images of fluorescence nanoparticles with and without a microsphere. The presence of a microsphere on top of the sample clearly enhanced the fluorescent signal. Figure 3(c) shows the fluorescent profile of 15 samples with (light blue lines) and without a microsphere (light red lines). The blue dashed line and the red dashed line show the average intensity of the 15 samples. The fluorescent enhancement factor, which is defined as the ratio of the averaged intensity of the fluorescence with a microsphere to that without a microsphere, is found to be ~5 (Figure 3(c)).

### Bidirectional acoustofluidic scanning of microspheres

**Fig. 4.**
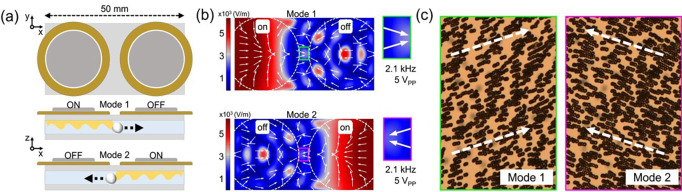
Acoustofluidic scanning device for bidirectional microsphere manipulation. (a) Schematic of the bidirectional acoustofluidic manipulation device, which incorporated two circular piezoelectric transducers bonded onto a cover glass with a thickness of 150 μm. The space between the two transducers was 6 mm. Mode 1 operates by pushing microspheres to the right; in contrast, mode 2 pushes them to the left. (b) Simulation results of the bidirectional acoustofluidic device in mode 1 and 2. The working frequency was 2.1 kHz and the amplitude was 4 V_PP_. The bidirectional acoustic streaming seen in the two modes used were shown in the green and pink boxes found in the working area. (c) Stacked microscopic images from the space between two transducers show that 20 μm microspheres were manipulated bidirectionally by acoustic streaming in each operation mode.

To perform the efficient 2D scanning process in a large field of view, we designed and fabricated a bidirectional acoustofluidic scanning device. As shown in Figure 4(a), two circular piezoelectric transducers were bonded onto a cover glass substrate with a thickness of 150 μm. A distance of 6 mm was present between the two transducers, meaning the detection area size was sufficient for microscopic imaging even under the largest field of view of a 4x objective lens which is typically 4 mm in diameter. This bidirectional acoustofluidic scanning design can operate in two modes programmed in a MATLAB interface that allow us to control the direction of acoustic wave propagation and the scan direction of the microspheres separately.

Figure 4(b) shows the simulated acoustic energy distribution under the operation of mode 1 and mode 2. The simulation assumes an electric wave of 2.1 kHz with a peak-to-peak voltage of 4 V_PP_ is applied on the transducer. The insets show the acoustic energy distribution between the two piezoelectric transducers with the white arrows showing the direction of the acoustic energy flow. Figure 4(c) shows the stacked experimental images of microsphere movement under the operation of mode 1 and mode 2. The microspheres are scanned from left to right and from right to left in mode 1 and mode 2, respectively (see a video in supplementary video SV1). Compared to the acoustic scan with one transducer in our previous work,^34,35^ this bidirectional scanning design gives us more degrees of freedom to scan the microspheres and achieve high-resolution, large-field-of-view images.

### Image distortion correction and large-field-of-view imaging

**Fig. 5.**
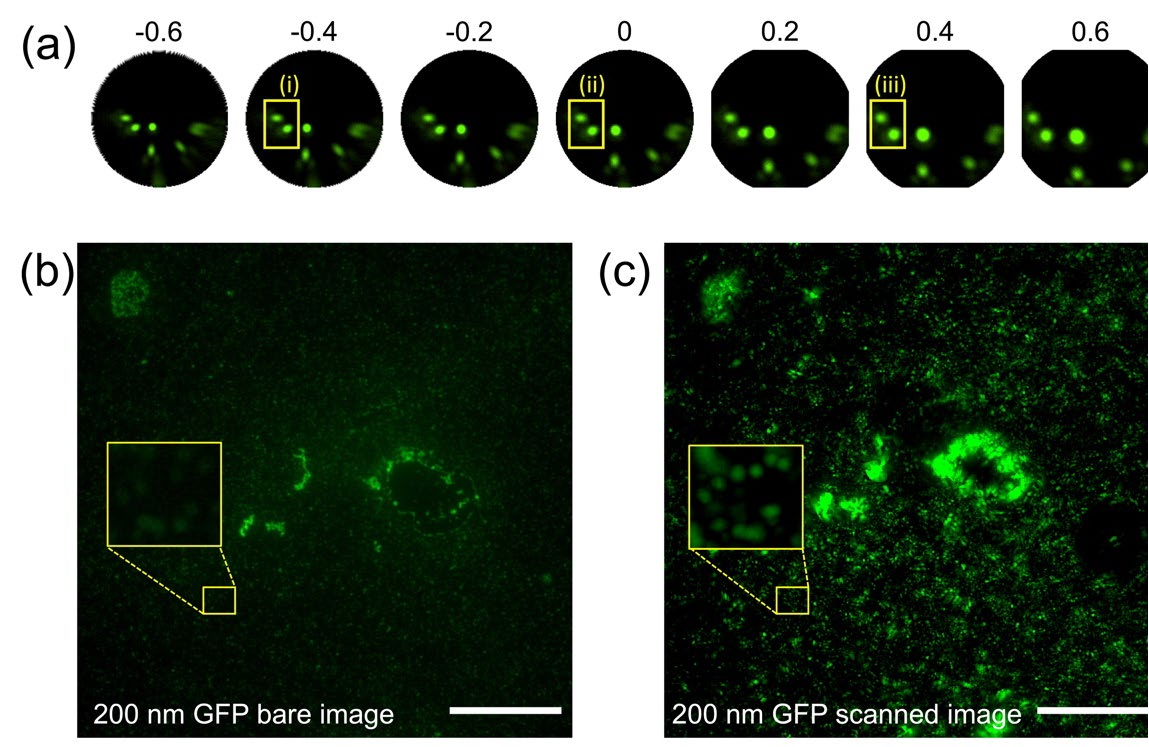
Image restoration of lens distortion and large field of view imaging in the acoustofluidic scanning fluorescence nanoscope. (a) Image distortion restoration with different parameters. A parameter of 0 indicates the original image. A negative parameter resulted in a correction of a barrel distortion, and a positive parameter resulted in a correction of a pincushion distortion. A barrel type distortion was observed in the small yellow box in the original image (ii). The −0.4 adjusted image (i) displayed a distorted nanoparticle image, the 0.4 adjusted image (iii) showed round nanoparticles due to the distortion being corrected. (b) A bare image of 200 nm nanoparticles deposited onto the sample surface. (c) An acoustofluidic scanned image of the same sample. 141 scanned images were processed to create the final scanned image. The yellow boxes indicate the same region of interest on the sample. The scale bar is 50 μm.

The off-axis fluorescent image from a microsphere suffers large image aberrations as manifested in the image (the comet-like tails in the images located at the edge of the microsphere) shown in Figure 3(b). Since each of the distorted images comes from a single nanoparticle, its distortion is corrected by a Matlab algorithm that allows us to adjust different types of lens distortion by changing the value of an input parameter. Figure 5(a) shows the effect of different values of the parameters on the image correction. The original image (ii) is corrected by assigning a positive value of 0.4 as shown in image (iii) in Figure 5(a). In contrast, a negative value of −0.4 deteriorated the image. To further verify this image correction process, a more identifiable sample (a line grating) is imaged by a microsphere and the image of the grating can be successfully recovered by using this image correction process as shown in Supplementary Figure S2.

After confirming the image correction process, a large field-of-view fluorescent image can be obtained by merging the images from each scanning microsphere and applying the image-correction algorithm. A Python image processing tool is used to merge the images. The location data of each image is first generated in camera #2 and then applied to the image data in camera #1. Each microsphere image was then cropped and pasted to create the final scanned image. This procedure was executed recursively until every region was covered.

Figure 5(b) shows the microscope image of 200 nm green fluorescence nanoparticles obtained directly from a microscope without the use of microspheres. Figure 5(c) demonstrates the same image obtained from scanning microspheres, which shows detailed nanoparticle samples with stronger fluorescence intensity. The yellow boxes in figure 5(b,c) indicate a much higher intensity in the same region of interest in the scanned image. To achieve the fully scanned image (200 × 200 μm field of view), we acquired and processed 141 microsphere images within 5 min (1 min of image acquisition, 4 min of image processing time).

## CONCLUSION

In this work, we developed an acoustofluidic scanning fluorescence nanoscope that can achieve superior resolution without sacrificing the field of view of the image. In contrast, a trade-off has to be made between resolution and field of view for most conventional fluorescence microscopes. The presence of a microsphere in the scanning fluorescence nanoscope lead to enhanced fluorescence compared to that without a microsphere. The bidirectional acoustofluidic scanning design allowed excellent freedom to control the scan of the microsphere. The dual-camera configuration enabled us to collect the fluorescent signal as well as the position information of each microsphere to form the image with a large field of view. Finally, the image-correction algorithm significantly reduced image distortion, resulting in a clearer and more accurate representation of the sample. Based on these features, the acoustofluidic scanning fluorescence nanoscope can be valuable for biomedical imaging and lab-on-a-chip systems.

## EXPERIMENTAL SECTION

### Optical characterization

As shown in Figure 1(c), we installed a CMOS camera (Zyla 4.2 Plus, Andor, USA) for fluorescence imaging and a CMOS camera (DFK 33UX264, Imagingsource, USA) for microsphere tracking on an upright microscope (BX51WI, Olympus, Japan) with a 60x objective lens (NA: 0.7, Olympus, Japan). A white light source was combined with a blue bandpass filter (FL488-10, Thorlabs, USA) to act as an excitation source, and the fluorescence camera was combined with a green bandpass filter (FB530-10, Thorlabs, USA) to receive the fluorescence light from the sample. To capture both images simultaneously, we installed a 50:50 beam splitter (CCM1-BS013, Thorlabs, USA) at the intersection point between the two cameras.

### Fabrication of the acoustofluidic device

Two circular piezoelectric transducers (AB2720B-LW100-R, PUI Audio, Inc., USA) were bonded onto a 150 μm thick cover glass (24×50 mm C8181-1PAK, Sigma–Aldrich, USA) with epoxy bonding (PermaPoxyTM 5 Minute General Purpose, Permatex, USA). The distance between the two transducers was 6 mm.

### Microsphere preparation and experimental setup

To perform the microsphere imaging, we chose 20 μm polystyrene microspheres (refractive index: 1.6, Sigma–Aldrich, USA). The microspheres were diluted with deionized water before being placed on the sample surface. To maintain a consistent water channel height between the device and sample, a square cover glass (#1.5, 10 × 10 mm, Ted Pella, USA) was placed at both ends of the device. The MATLAB (version: R2021) script was designed and executed to control the function generator (FY6600, FeelTech, China) and CMOS cameras simultaneously. These cameras were used to collect the image data. An acoustic burst mode with 0.2 second intervals was applied, and the image acquisition for the two cameras was executed every 0.2 seconds.

### Simulation of the acoustic field

To understand the acoustic energy distribution within the device, a model of an acoustic device was designed in COMSOL Multiphysics^®^. The model included two piezoelectric transducers, a cover glass, and water under the cover glass. A time domain study was used to visualize the transducer excitation. A 2.1 kHz and 4 V_PP_ signal was applied to the transducer using the electrostatics module. A low reflection boundary water layer with open channel conditions was applied to the cover glass layer. We observed the vibration profile and acoustic streaming to determine the proper microsphere manipulation area, which was the space between the two transducers.

### Imaging sample preparation

To experimentally demonstrate the scanning performance of the system, we fabricated a fluorescence nanoparticle sample with a hard PDMS (PP2-RG07, Gelest, Inc., USA) membrane. The green fluorescence nanoparticle sample (200 nm: FSDG002, 500 nm: FSDG003, Bangs Laboratories, Inc., USA) was diluted with deionized water and loaded on the cover glass (24×50 mm C8181-1PAK, Sigma–Aldrich, USA). Then the sample was dried at room temperature for 3–6 hours. After drying, we applied a hard PDMS mixture to the sample and ran the spin-coat (WS-650-23, Laurell Technologies, USA) process. Then, the sample was baked at 60°C for 30 minutes in the oven.

### Image processing method

To generate the final scanned image, the collected images were processed in the following order. First, a circle-finding algorithm was executed on the image from Camera #2, as seen in the bottom panel of Figure 1(c), which stored information on the microsphere coordinates and radius. The magnification factor was calculated using the ratio of the sample grating line pitch length between Camera #1 and Camera #2. The calculated magnification factor (0.984) was then multiplied with the coordinates and radius information and applied to the images from Camera #1, as shown in the top panel of Figure 1(c). Next, the microsphere magnified circle images were cropped from the images of Camera #1. Finally, the cropped images were pasted onto the final image with a lens distortion restoration technique to ensure clear matching between the images. Each image was processed recursively in the same manner. The final scanned image was generated by the repetitive image processing algorithm.
